# The TRIPLEX study: use of patient-derived tumor organoids as an innovative tool for precision medicine in triple-negative breast cancer

**DOI:** 10.1186/s12885-023-11362-8

**Published:** 2023-09-19

**Authors:** Jordane Divoux, Romane Florent, Margaux Jacobs, Justine Lequesne, Jean-Michel Grellard, Chankannira San, Sara Grossi, Katia Kerdja, Bénédicte Clarisse, Gwenaelle Boudier, François Cherifi, Mélanie Briand, Enora Dolivet, Alisson Johnson, Brice Dubois, Valentin Harter, Joëlle Lacroix, Charlotte Raboutet, Brigitte Marie, Nathalie Rousseau, Cécile Blanc-Fournier, Dominique Vaur, Martin Figeac, Laurent Poulain, Louis-Bastien Weiswald, George Emile

**Affiliations:** 1 INSERM U1086 ANTICIPE (Interdisciplinary Research Unit for Cancers Prevention and Treatment), BioTICLA Laboratory (Precision Medicine for Ovarian Cancers), Université de Caen Normandie, Caen, France; 2 Comprehensive Cancer Center François Baclesse, UNICANCER, Caen, France; 3 US PLATON, ORGAPRED Core Facility, Université de Caen Normandie, Caen, France; 4 Comprehensive Cancer Center François Baclesse, Breast Cancer Unit, UNICANCER, Institut Normand du Sein, Caen, France; 5 Comprehensive Cancer Center François Baclesse, Clinical Research Department, UNICANCER, Caen, France; 6 US PLATON, Biological Resource Center ‘OvaRessources’, Université de Caen Normandie, Caen, France; 7 Comprehensive Cancer Center François Baclesse, Department of Surgery, UNICANCER, Caen, France; 8 Comprehensive Cancer Center François Baclesse, North-West Canceropole Data Center, UNICANCER, Caen, France; 9 Comprehensive Cancer Center François Baclesse, Department of Radiology, UNICANCER, Caen, France; 10 Biological Resource Center ‘Tumorotheque de Caen Basse-Normandie’, IRCBN Institut Régional du Cancer Basse Normandie, Caen, France; 11 Comprehensive Cancer Center François Baclesse, Department of Biopathology, UNICANCER, Caen, France; 12 Comprehensive Cancer Center François Baclesse, Department of Cancer Biology and Genetics, UNICANCER, Caen, France; 13 CNRS, Inserm, CHU Lille, Institut Pasteur de Lille, US 41 - UAR 2014 - PLBS, University of Lille, Lille, France; 14 INSERM U1086 ANTICIPE (Interdisciplinary Research Unit for Cancers Prevention and Treatment), Comprehensive Cancer Center François Baclesse, 3 Avenue du Général Harris, BP 45026, Caen Cedex 05, 14 076 France; 15 Breast Cancer Unit, Comprehensive Cancer Center François Baclesse, 3 Avenue du Général Harris, BP 45026, Caen Cedex 05, 14 076 France

**Keywords:** Triple negative breast cancer, Patient-derived tumor organoids, Predictive functional assays, Chemo-immunotherapy

## Abstract

**Background:**

Triple negative breast cancers (TNBC) account for approximately 15% of all breast cancers and are associated with a shorter median survival mainly due to locally advanced tumor and high risk of metastasis. The current neoadjuvant treatment for TNBC consists of a regimen of immune checkpoint blocker and chemotherapy (chemo-ICB). Despite the frequent use of this combination for TNBC treatment, moderate results are observed and its clinical benefit in TNBC remains difficult to predict. Patient-derived tumor organoids (PDTO) are 3D in vitro cellular structures obtained from patient’s tumor samples. More and more evidence suggest that these models could predict the response of the tumor from which they are derived. PDTO may thus be used as a tool to predict chemo-ICB efficacy in TNBC patients.

**Method:**

The TRIPLEX study is a single-center observational study conducted to investigate the feasibility of generating PDTO from TNBC and to evaluate their ability to predict clinical response. PDTO will be obtained after the dissociation of biopsies and embedding into extra cellular matrix. PDTO will be cultured in a medium supplemented with growth factors and signal pathway inhibitors. Molecular and histological analyses will be performed on established PDTO lines to validate their phenotypic proximity with the original tumor. Response of PDTO to chemo-ICB will be assessed using co-cultures with autologous immune cells collected from patient blood samples. PDTO response will finally be compared with the response of the patient to evaluate the predictive potential of the model.

**Discussion:**

This study will allow to assess the feasibility of using PDTO as predictive tools for the evaluation of the response of TNBC patients to treatments. In the event that PDTO could faithfully predict patient response in clinically relevant time frames, a prospective clinical trial could be designed to use PDTO to guide clinical decision. This study will also permit the establishment of a living biobank of TNBC PDTO usable for future innovative strategies evaluation.

**Trial registration:**

The clinical trial (version 1.2) has been validated by local research ethic committee on December 30^th^ 2021 and registered at ClinicalTrials.gov with the identifier NCT05404321 on June 3^rd^ 2022, version 1.2.

## Background

### Breast cancer: epidemiology and therapeutic management

Breast cancer is the most common cancer worldwide and accounts for 1 in 8 cancer diagnoses with a total of 2.3 million new cases in both sexes combined [1]. It represents about a quarter of all cancer cases in females and was by far the most commonly diagnosed cancer in women in 2020 [2]. An estimated 685,000 women died worldwide from breast cancer in 2020, corresponding to 16% of cancer deaths in women [3]. Classification of breast cancer is done through the analysis of the expression of hormone receptors (estrogen/progesterone) and human epidermal growth factor receptor 2 (HER2). They are regrouped as hormone receptors-positive, HER2 positive or triple negative. Triple negative breast cancer (TNBC) represents about 15% of all breast cancers, it occurs generally in younger patients and is associated with a higher risk of metastasis and worse survival [4]. These tumors are more likely to be locally advanced and require neoadjuvant treatment [5]. The current neoadjuvant regimen for TNBC is based on the KEYNOTE-522 trial and consists of four cycles of an immune checkpoint blocker (ICB), pembrolizumab every 3 weeks plus paclitaxel and carboplatin, followed by 4 cycles of pembrolizumab plus doxorubicin–cyclophosphamide or epirubicin–cyclophosphamide [6], two regimens summarized as chemo-ICB in the following text. Based on the same study, the post neoadjuvant treatment for TNBC consists of the continuation of pembrolizumab for 9 additional cycles. ICB is thus nowadays a major therapeutic option for TNBC care although other adjuvant treatments can be used such as capecitabine for 6 to 8 cycles [7] or olaparib for patients with a BRCA 1 or 2 mutation [8].

Despite the frequent use of chemo-ICB for TNBC treatment, moderate results continue to be observed for this regimen and the clinical benefit of ICB in TNBC remains hard to predict. This is explained by the fact that patients show highly variable response with a minority of good responders currently not clearly identified. To date, only one biomarker is clinically validated to predict ICB efficacy in TNBC and consists in the evaluation of PD-L1 expression (FDA 2020). However, despite its predictive value in advanced TNBC, PD-L1 expression failed to discriminate responding and non-responding patients in early TNBC, in which ICB efficacy seems to be higher [9]. Other biomarkers are currently considered such as tumor mutational burden (TMB), tumor infiltrating leucocytes (TILs) or immune genes signatures but their predictive value is still controversial and their use is for now restricted to general prognosis [9]. The development of an efficient test to predict chemo-ICB efficacy in early TNBC is thus a prerequisite to make the best use of it and drive TNBC patients care toward precision medicine.

### Patient derived tumor organoid (PDTO): an innovative tool for precision medicine

The essence of precision medicine in oncology is to give the right treatment to the right patient, i.e. to the patient which will have the best chance to benefit from a given treatment. So far, the predominant method to reach this goal was to select the treatment based on biomarkers able to discriminate responder from non-responder patient but today there are growing strategies focusing on functional assays [10]. These assays are based on direct exposure of cancer tissues derived from patients to drugs to evaluate the response to treatments. They thus assume the tumor on its all and consider all its features (histological and molecular) without any need of prior characterization. Different models can be used to run functional assays [11], such as Patient-Derived Tumor Organoids (PDTO) which are now considered as a relevant model to bridge the gap between cell lines and patient-derived xenograft mouse models. PDTO are three-dimensional in vitro cellular structures obtained after dissociation of tumor samples and embedding of tumor cells in extracellular matrix within medium containing a cocktail of growth factors and signaling pathway inhibitors. Long-term PDTO lines displaying similar morphologic and genetic features with their original tumor have been successfully established from a range of malignancies [12], including breast cancer [13]. Increasing evidence indicate that the ex vivo response of PDTO may correlate with the response of the original tumor. PDTO have thus been shown to be able to predict the response to chemotherapy of gastrointestinal [14] and pancreatic tumors [15] as well as the sensitivity of colorectal cancers to radiotherapy [16]. Furthermore, two independent studies showed that PDTO established from different types of breast cancers, including TNBC, displayed histological and genomic concordance with parental tumors as well as sensitivities to standard of care corresponding to their tumor type (chemotherapy, hormonotherapy and HER2 antibodies) [17, 18]. Given the predictive value of PDTO, their use to predict chemo-ICB efficacy against TNBC may have promising potential for the future. Nevertheless, as immunotherapy efficacy requires the presence of a functional immune system, enhancement of PDTO with autologous immune cells (iPDTO) is critical and elicits a significant interest from the scientific community. Several attempts have been described to generate iPDTO either by using immune cells infiltrating the tumor of origin [19–26] or from peripheral blood mononuclear cells (PBMC) [27–29]. Still, coculture of PDTO and immune cells is facing a number of challenges which will need to be addressed before the availability an off-the-shelf predictive tool.

## Method/design

**Fig. 1:**
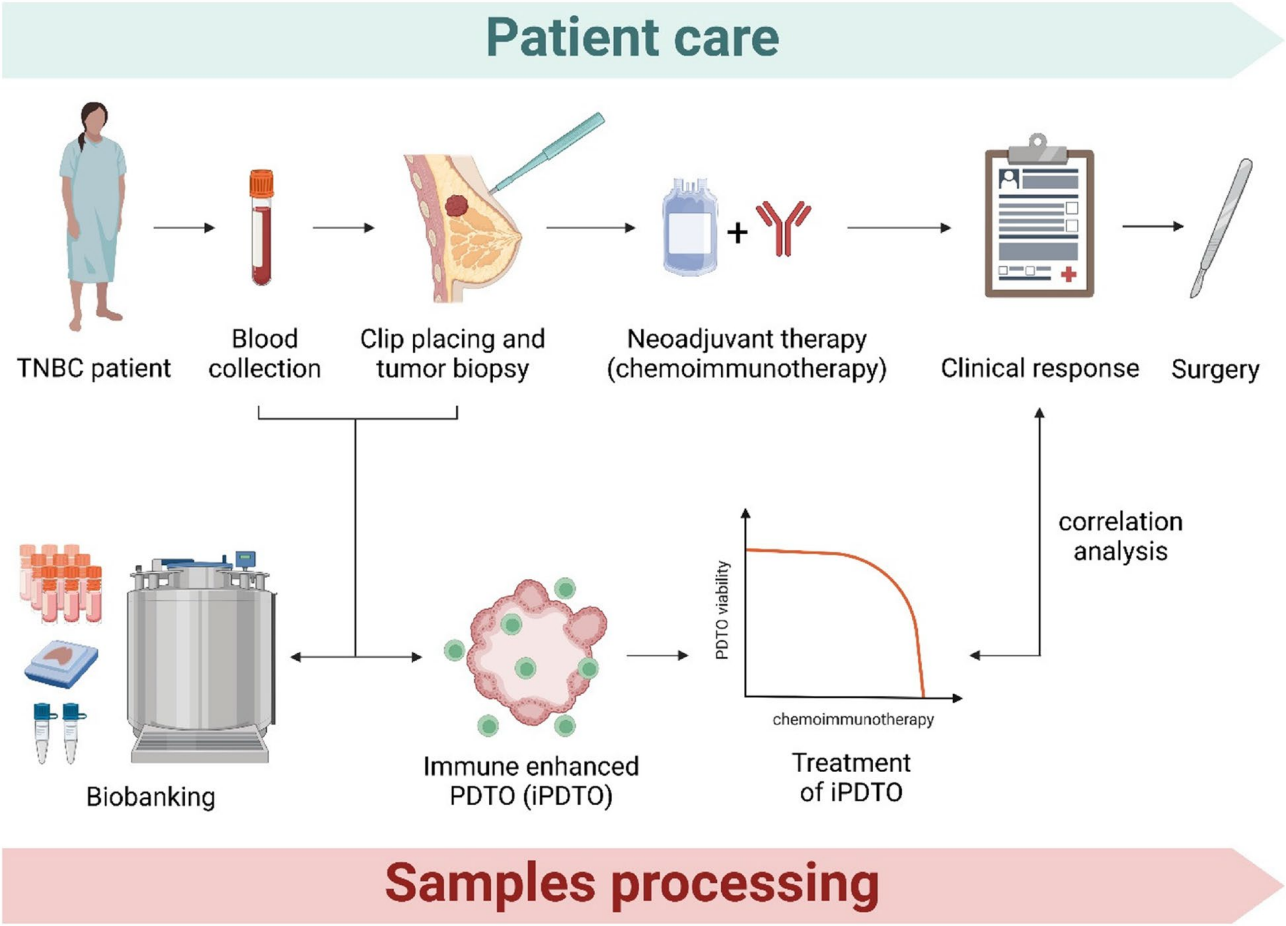
TRIPLEX study design (created with Biorender.com)

The TRIPLEX study is a single-center observational study conducted at Comprehensive Cancer Centre François Baclesse to investigate the feasibility of generating and testing PDTO from TNBC for evaluation of response to treatments (Fig. 1). The TRIPLEX study and this manuscript have been written in accordance with standard protocol items, namely recommendations for interventional trials (SPIRIT). The method and design of this study is based on the ORGAVADS study described by Perréard et al. [30].

### Study objectives and endpoints

The main objective of the study is to assess the feasibility of using PDTO from TNBC as a tool for predicting response to treatments.

The secondary objectives are: i) to assess the feasibility of in vitro functional assays for evaluation of sensitivity to treatments; ii) to identify biomarkers for predicting response to treatments in tumor and blood samples; iii) to evaluate the concordance between the response of PDTO to treatments (chemotherapy, targeted therapies and immunotherapy) and the clinical response.

### Study population

Eligibility criteria are described in Table 1. The TRIPLEX study focuses on patients with early stage (I-III) TNBC who undergo clip placement before neoadjuvant chemotherapy at Comprehensive Cancer Centre François Baclesse.

**Table 1:** TRIPLEX study inclusion and exclusion criteria

Inclusion criteria	Exclusion criteria
Patient over 18 years of age	Pregnant women
Patient with early stage (I-III) TNBC who needs to have clips placed before neoadjuvant chemotherapy	Persons deprived of liberty or under guardianship (including curatorship)
Patient affiliated to a social security system	History of any other clinically active malignancy in the last 5 years prior to inclusion
Proficiency in French language	
Patient having signed the consent to participate in the study	

### Study assessment

The study was approved by the “North-West I” ethical committee (IDRCB: 2021-A02676-35). After the screening of patients according to criteria, a proposal for enrollment will be given by the clinician which will inform all patients enrolled in the study that their biological samples could be used for research purposes. An identification number will be assigned to each patient to be used throughout the study. All patients participating may object at any time, leading to the prompt disposal of their tissue and any derived material, as well as the cessation of data collection. The enrollment period of the study will be four years.

### Medical data collection

In order to correlate the biological data obtained on the initial tumor with the response to ex vivo treatments and the response observed in the clinic, the patients' clinical data will be collected in the study from medical records. The collected data are summarized in Table 2.

**Table 2:** Medical data collected in the TRIPLEX study

Clinical parameters
Age
History of the disease (diagnosis, mutations status, management)
History of other cancers or not
Surgical procedure
Response to treatment
Recurrence (type, date, location)
Date of death

### Biological collection

#### Tumor

During the patient’s care, a breast clip is placed under local anesthesia at the site of the tumor in order to locate it for its excision after the neoadjuvant treatment. During this medical act, three tumor samples will be collected through a core biopsy. Tumor samples will be then sent directly in sterile vials filled with cold culture medium supplemented with a Rho-kinase inhibitor (Y-27632) to the laboratory.

#### Blood

Blood samples are collected before the clip placement as part of regular medical care. No blood draw will be done specifically for this study. An additional volume of blood will be collected in seven 5 ml EDTA tubes and transported to the laboratories to be processed.

### Biological sample processing

#### Tumor sample processing

Different procedures will be carried out on the three tumor samples: 1) one sample will be processed for the isolation of viable cells and PDTO establishment; 2) one sample will be snap frozen and stored at -80 °C for molecular analyses; 3) one sample will be fixed in paraformaldehyde for paraffin embedding and subsequent histopathological analysis and immunohistochemistry. All tumor samples will be stored in the Biological Resource Center (BRC) ‘Tumorotheque de Caen Basse-Normandie’ (TCBN).

#### Isolation of PBMC

PBMC will be isolated from blood by density gradient centrifugation using Ficoll-Paque in Leucosep tubes. Cells will be resuspended in cold culture media, and counted. PBMC will be then resuspended in freezing solution (10% DMSO, 90% FBS), aliquoted (about 5 cryovials, 4.10^6^ cells/cryovial), and frozen with gradually decreasing temperatures (1ºC/min) to -80ºC before long-term storage at liquid nitrogen temperatures and stored in the BRC TCBN.

#### Establishment of panel of PDTO

Tumor samples will be enzymatically and mechanically dissociated to obtain isolated cells or small cell clusters. Cells will be embedded in an extracellular matrix and cultured in a medium supplemented with growth factors and signal pathway inhibitors [Advanced DMEM (Gibco) supplemented with 100 UI/mL of penicillin and streptomycin (Gibco), 1% GlutaMAX (Gibco), 1X B27 (Gibco), 5 mM Nicotinamide (Sigma-Aldrich), 1.25 mM N-Acetyl-L-Cysteine (Sigma-Aldrich), 50 μg/mL Primocin (InvivoGen), 100 ng/mL Noggin (Peprotech), 5 nM Neuregulin 1 (Peprotech), 5 μM Y27632 (Interchim), 20 ng/mL FGF-10 (PeproTech), 500 nM A-83–01 (PeproTech), 50 ng/mL EGF (PeproTech), 5 ng/ml FGF-7 (PeproTech), 1 µM SB202190 (PeproTech) and 10% RSPO1- conditioned media (Cultrex HA-R-Spondin-1-Fc 293 T, Amsbio)]. Culture medium will be changed twice a week. Once formed, PDTO will be dissociated and reseeded to amplify them for experimental purposes. Cryovials will be prepared at regular intervals by dissociating and resuspending PDTO in Recovery Cell Culture Freezing Medium (Gibco) prior to be biobanked in liquid nitrogen. PDTO lines will be considered as established when it will be maintained for more than 3 passages. For each established PDTO line, samples will be kept frozen for DNA/RNA/protein analysis and others will be embedded in paraffin for histopathological analysis.

#### Coculture of PDTO with immune cells

PDTO specific autologous T cells will be induced according to modified version of the protocol described in Dijkstra et al. [29]. Briefly, PBMC will be activated with the corresponding PDTO lysate and specific T cells clones will be isolated based on their expression of CD154 and CD137 markers using flow cytometry sorting. Once isolated and their purity controlled, specific T cells will be amplified by the use of a stimulation matrix and then cryopreserved. A quality control will be performed before cryopreservation by flow cytometry to check for reactivity against PDTO using CD107a expression and cytokines production after antigen re-stimulation. Once produced and checked for antigen specificity, PDTO-specific T cells will be cocultured with PDTO to produce iPDTO for the evaluation of response to immunotherapy.

#### Evaluation of the response to treatment and correlation with clinical data

**Fig. 2:**
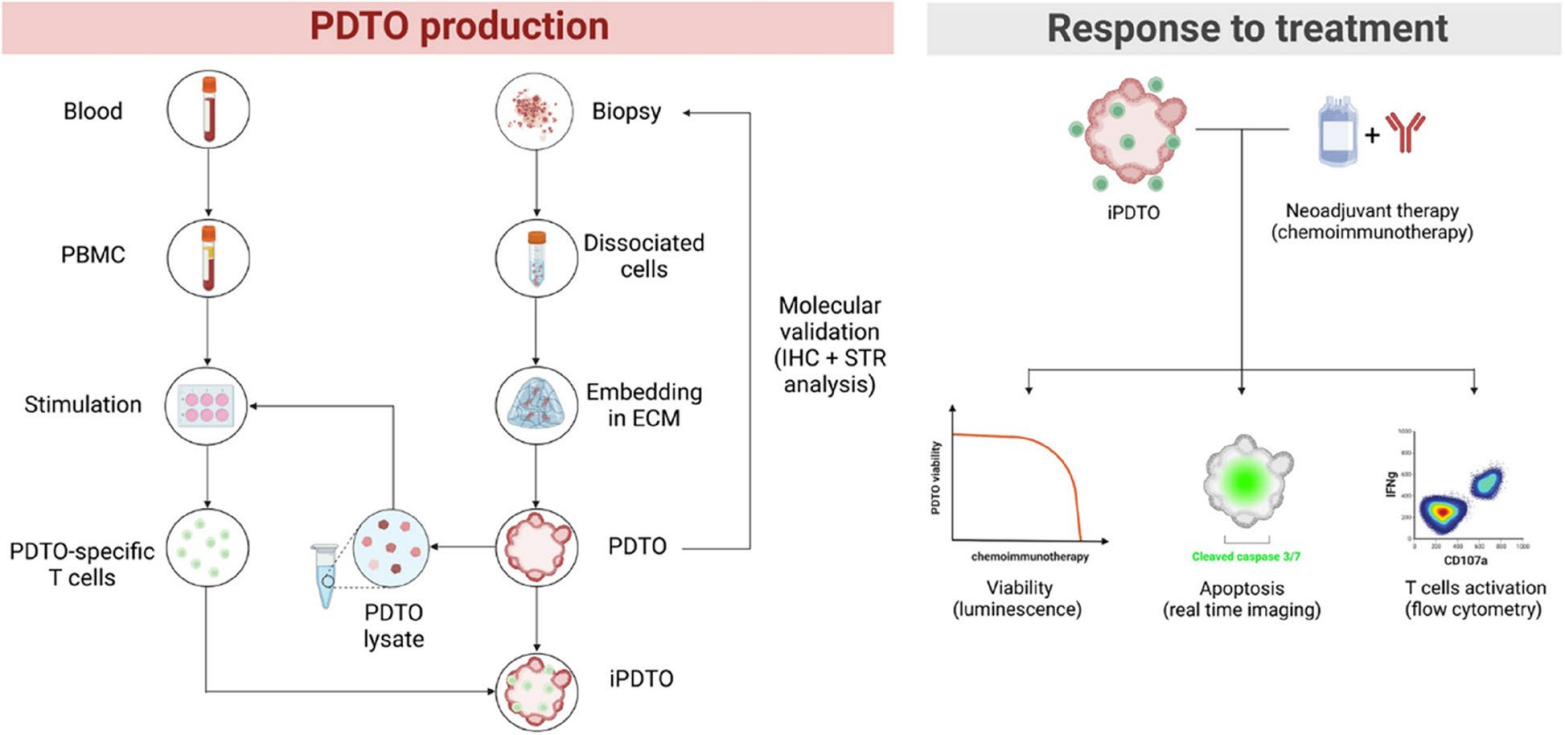
PDTO generation and treatment (created with Biorender.com)

iPDTO will be done at several effector:target ratios to determine the sensitivity of PDTO to T cells killing. Viability of PDTO will be evaluated all along the assay thanks to the use of Caspase 3 fluorescent probes imaged by IncuCyte (Sartorius). The phenotype of T cells (CD107a expression and cytokines production) will be evaluated at the end of the coculture by flow cytometry. The process will be repeated in the presence of a combination of chemotherapy and ICB to evaluate the impact of the treatment (Fig. 2). This information will be re summarized in the “immune sensitivity” and the “chemo-ICB sensitivity” scores that will be compared with the patient’s clinical response to assess the predictive capacity of the model. The clinical response of the patient will be evaluated through a Residual Cancer Burden (RCB) score established by immunochemistry. Clinical responses will be classified as RCB-0 or pCR (Pathological Complete Response), RCB-I (minimal residual disease), RCB-II (moderate residual disease) and RCB-III (extensive residual disease).

### Evaluation of PDTO model relevance and identification of potential predictive biomarkers

#### Transcriptomic analysis

RNA analysis will be performed according to the protocol described in Perréard et al. [30]. Briefly, total RNA will be extracted using the Nucleospin RNA kit (Macherey Nagel, Hoerdt) and libraries will be made with the QuantSeq 3’RNA Library Kitto. Once produced, the final library will be purified and deposed on High sensitivity DNA chip to be controlled on Agilent bioanalyzer 2100 and sequenced on NovaSeq 6000 (Illumina). Elimination of poor-quality regions and poly(A) of reads will be done through the use of the fastpq program. Read alignments will be performed using the program STAR with the genome reference human (GRCh38) and the reference gene annotations (Ensembl). Reads counts will be done using FeatureCount and statistical analysis will be realized with the R/bioconductor package DESeq2.

#### Copy number variations analysis by low-pass whole genome sequencing (WGS)

WGS will be performed using Illumina DNA PCR Free prep kit, starting with 500ng of DNA. Library will be prepared with HMMcopy and ichorCNA.

#### Transcriptome and CNV analysis

Analysis of intra reproducibility and differences between original tumors and PDTO will be assessed by PCA and unsupervised hierarchical clustering as described in Perréard et al. [30].

### Statistical consideration

#### Sample size determination

To estimate the PDTO establishment rate, assumed around 30%, with a 95% confidence interval of 10% width, 141 tumor samples will be required. Anticipating 15% of non-assessable samples, it is planned to include 163 patients. We expect to be able to correlate the clinical response with the response to the treatments obtained ex vivo on about 30% of included patients, namely 49 PDTO. With such a sample, the disagreement rate between clinical response and ex vivo response will be estimated with a 95% confidence interval of 20% width (estimating this disagreement rate around 15%).

#### Statistical analyses

Qualitative variables will be described using the sample numbers and percentages. Quantitative variables will be described using the mean (± standard deviation) or the median and the range if normality hypothesis is not verified*.*

To address the primary objective, the rate of successful PDTO establishment, i.e., the rate of tumor samples usable for predictive functional assays based on PDTO, will be estimated with its 95% confidence interval. Then, PDTO response to treatment will be correlated with the clinical response by computing the Cohen’s kappa coefficient. Associations between biological parameters and clinical response will be assessed by one-way analysis of variance (or the non-parametric Kruskal–Wallis test, if necessary). Receiver Operating Characteristic (ROC) curves and a logistic regression model will also be used to identify predictive factors of clinical response. An alpha level of 5% will be considered to indicate statistical significance for each statistical analysis and confidence interval.

### Data management

The tumor collections of the BRC TCBN are associated to database, where information about the patient, the histology and pathology of the tumor and the treatment are stored. In the frame of this present application, follow-up data of the patients included in the study (occurrence of local or locoregional relapse, distant metastasis, second primary cancer, death after ICB treatment) will be retrieved from medical charts by a Clinical Research Associate. Physicians will regularly confirm relapse and/or distant metastasis by reviewing radiological examination reports.

## Discussion

PDTO are preclinical models that recapitulate closely the original tumors in terms of morphological and molecular characteristics [31]. Recent studies have demonstrated that PDTO could potentially mirror the clinical responses of patients to treatments but the sample size of most studies is too small or too heterogenous to clearly conclude about its predictive value in clinical practice [12]. In particular, the concordance between response of PDTO and response of breast cancer patient to tamoxifen was studied in only 2 patients [13]. It is therefore crucial to demonstrate the predictive value of PDTO based on large and homogenous patient cohort.

In this clinical study, we propose to establish PDTO from biopsies of TNBC patients who will undergo neoadjuvant therapy. These biopsies will be collected at the tissue marker clip placement. This allows the direct comparison between the response of the patient after neoadjuvant treatment (assessed by Residual Cancer Burden (RCB)) and the response of PDTO after exposure to the same treatment. Our goal is to show that PDTO from TNBC patients could serve as a powerful tool for predicting patient response to treatments and to identify predictive molecular signature in PDTO, tumor and blood samples. An additional aim is to use the PDTO generated during this study to assess new therapeutic compounds and strategies. In the event that PDTO could faithfully predict patient response in clinically relevant time frames, a prospective clinical trial could be designed to use PDTO for guiding neoadjuvant therapy clinical decision making.

Another perspective will be to generate PDTO from biopsies of recurrent breast cancers to help clinicians determine the most optimal therapy. In this manner, it is crucial to collect biopsies at the right time to take into consideration the genetic and epigenetic changes associated with the first line of treatment. Furthermore, a comparison of the response of the PDTO obtained at two different times (early and recurrent) may also inform on the predictive potential of PDTO derived from early biopsies to predict the response to following lines of treatments.

## Data Availability

Not applicable.

## Abbreviations

BRC: Biological Resource Center
BRCA: BReast CAncer
CD: Cluster Differentiation
CNV: Copy Number Variation
DMEM: Dulbecco’s Modified Eagle Medium
DMSO: DiMethyl SulfOxide
DNA: DeoxyriboNucleic Acid
EDTA: EthyleneDiamineTetraacetic Acid
EGF: Epidermal Growth Factor
ER: Estrogen Receptor
FBS: Fetal Bovine Serum
FGF: Fibroblast Growth Factor
HER2: Human Epidermal Growth Factor Receptor-2
ICB: Immune Checkpoint Blockade
iPDTO: Immune enhanced PDTO
PBMC: Peripheral Blood Mononuclear Cells
PCA: Principal Component Analysis
PD-L1: Programmed Death-Ligand 1
PDTO: Patient-Derived Tumor Organoid
PR: Progesterone Receptor
RCB: Residual Cancer Burden
RNA: RiboNucleic Acid
ROC: Receiver Operating Characteristic
RSPO1: R-SPOndin-1
SPIRIT: Standard Protocol Items: Recommendations for Interventional Trials
TCBN: “Tumorothèque de Caen Basse Normandie»
TILs: Tumor Infiltrating Leucocytes
TMB: Tumor Mutational Burden
TNBC: Triple Negative Breast Cancer
TRIPLEX: TRIPLe-negative cancer breast EXvivo
WGS: Whole Genome Sequencing
